# On Route to Chimeric Antigen Receptor T-cell (CAR T) Therapy, Less Is More: Adaptive Bridging Radiotherapy in Large B-cell Lymphoma

**DOI:** 10.7759/cureus.67572

**Published:** 2024-08-23

**Authors:** Hazim Ababneh, Mislav Bobić, Jennifer Pursley, Chirayu Patel

**Affiliations:** 1 Radiation Oncology, Massachusetts General Hospital, Harvard Medical School, Boston, USA

**Keywords:** bridging therapy, ethos, car t-cell therapy, large b-cell lymphoma, adaptive radiation therapy

## Abstract

CD19-targeted chimeric antigen receptor (CAR) T-cell therapy has appreciably advanced treatment for relapsed or refractory large B-cell lymphoma (LBCL). During the critical interim of four to six weeks, until CAR T-cells are ready, radiation therapy (RT) can be used to control the disease. We present the case of a 64-year-old female with relapsed/refractory diffuse large B-cell lymphoma (DLBCL) who received adaptive RT for bilateral adrenal masses as a bridging strategy before undergoing CAR T-cell therapy and enrolled in an adaptive RT clinical trial. A plan was developed to deliver up to five once-weekly fractions (5 Gy per fraction) of CT-based online adaptive RT (Varian Ethos with HyperSight imaging, Varian Medical Systems, Palo Alto, CA). The patient experienced rapid symptomatic relief, with no RT-related toxicities. The patient received RT at only half of the sessions (two out of four sessions) due to excellent tumor shrinkage on cone-beam CT (CBCT). As such, the patient was treated at a lower total dose (10 Gy) than she otherwise would have received with standard RT. Post-RT PET/CT showed significant disease regression, compatible with partial response, prior to CAR T-cell infusion. This case shows the successful application of adaptive RT as bridging therapy prior to CAR T-cell therapy, and we expect the results of this adaptive RT trial to guide the future of adaptive RT in relapsed/refractory B-cell lymphomas.

## Introduction

CD19-targeted chimeric antigen receptor (CAR) T-cell therapy has markedly shifted the treatment landscape for relapsed or refractory large B-cell lymphoma (LBCL), with impressive response rates and long-term survival rates of 42% at five years [1]. The eligibility criteria for CAR T-cell therapy continue to broaden, cementing its role in the curative treatment of multiple relapsed or refractory hematologic malignancies.

The time interval between the determination of eligibility for CAR T-cell therapy and the infusion - typically spanning four to six weeks - presents challenges in managing disease progression [2]. During this critical interim, the use of radiation therapy (RT) as a bridging therapy, either alone or in combination with systemic therapy, has been successful for select patients in controlling the disease by tumor debulking and/or maintaining adequate performance status by preventing the progression of potentially symptomatic disease, which is important in getting those patients to CAR T-cell therapy [2]. This approach has been effective in achieving local tumor control without amplifying the toxicities of CAR T-cell therapy infusion [2,3].

Bridging radiation to date has consisted of daily radiation treatments in an era in which real-time replanning was impossible. There is a lack of consensus on optimal dose fractionation, although hypofractionated regimens dominate [2]. Taken together with the fact that even refractory lymphoma is radiosensitive, leading to dramatic changes in size over time, the advent of adaptive RT allows the investigation of a new strategy of once-weekly hypofractionated radiation for refractory lymphoma. Adaptive RT allows dynamic adjustment of the radiation plan at each treatment fraction [4,5]. This adjustment is driven by real-time imaging, which captures any anatomical changes in the tumor, thereby optimizing tumor control while minimizing damage to surrounding healthy tissue. Adaptive RT plans are refined in real time, leveraging artificial intelligence (AI) to enhance precision under the close supervision of physicians and medical physicists.

While such adaptive RT approaches have been explored in solid tumors, studies focusing on their application in relapsed/refractory lymphoma, particularly in the bridging setting prior to CAR T-cell therapy, remain unexplored [6]. Thus, we present a case of a patient with relapsed/refractory diffuse large B-cell lymphoma (DLBCL) who received adaptive RT for bilateral adrenal masses as a bridging strategy before undergoing CAR T-cell therapy, enrolled in a clinical trial (NCT06004167, clinicaltrials.gov).

## Case presentation

**Figure 1:**
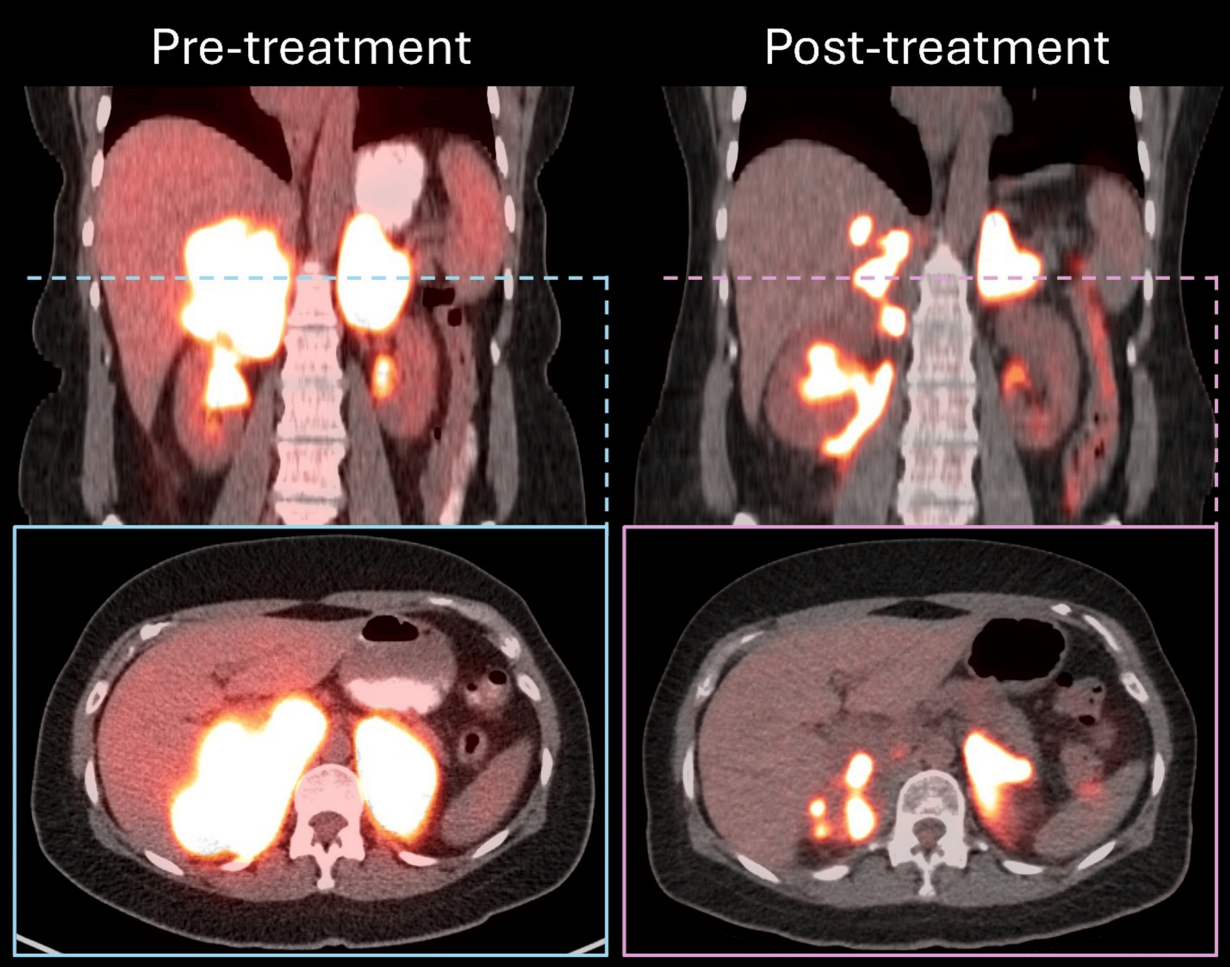
Pre-treatment and post-treatment PET/CT scans, showing the response to online adaptive radiotherapy.

The patient is a 64-year-old female with stage I, non-germinal center B-cell (non-GCB) DLBCL (with MYC and BCL6 mutations) of the left masticator space - she received treatment nine years ago with R-CHOP (rituximab plus cyclophosphamide, doxorubicin, vincristine, and prednisone), high-dose methotrexate CNS prophylaxis, and consolidative radiation to 36 Gy in 18 fractions. Five years ago, the patient had a left para-aortic relapse - she achieved remission with R-ICE (rituximab, ifosfamide, carboplatin, and etoposide) followed by BEAM (carmustine, etoposide, cytarabine, and melphalan) autologous stem cell transplantation. One month prior to radiation oncology evaluation, she presented with bilateral flank pain and fatigue, with PET/CT on day 54 (CAR T infusion = day 0), showing bilateral adrenal masses (Figure 1). Biopsy confirmed relapsed DLBCL. CAR T-cell therapy was recommended as a curative treatment strategy.

Given the delay from apheresis (day 40) to infusion of CAR T-cells, the patient needed bridging therapy. Multidisciplinary discussion recommended radiotherapy alone as bridging therapy. Of note, the patient lived a long distance away from the hospital. A plan was developed for up to five once-weekly fractions (5 Gy per fraction) of CT-based online adaptive RT (Varian Ethos with HyperSight imaging, Varian Medical Systems, Palo Alto, CA [7]). The patient was simulated in the supine position using a wing board and a knee fix cushion for setup reproducibility. Helical CT scans with a 2.5 mm slice thickness were acquired with and without IV contrast; the scan with IV contrast was used for assistance with contouring the target, while the scan without contrast was used for planning. A single gross tumor volume (GTV) for the bilateral adrenal masses was contoured with 0 mm PTV. Abdominal compression was not employed due to patient discomfort. Breath-hold equipment was not available for adaptive radiation. A four-dimensional CT (4DCT) was not used as target volumes needed to be immediately submitted after the CT simulation due to the urgent treatment timeline with the goal of bridging rather than definitive therapy. Given the bulk of the adrenal masses making complete geometric miss of disease unlikely, the goal of cytoreduction rather than definitive control, concerns of providing excess hypofractionated RT to the bilateral kidneys, and ability to follow response to treatment and adjust margins if required at future adaptive sessions, a 0 mm margin for PTV was used, departing from conventional standards developed for non-adaptive treatments for non-bridging radiation.

The following plan directives were specified: 99% of GTV coverage by 2,475 cGy with hot spot <108%; renal hilum V23 <15 cc (each); kidney (cortex) V18 <200 cc (total); liver V21 <700 cc; stomach max 26 Gy; pancreas mean 10 Gy; spleen mean 5 Gy; bowel V20 <30 cc, and spinal cord V22<0.35 cc. An intensity-modulated radiotherapy (IMRT) plan with nine equidistant fields was developed in the Ethos planning system. The optimization goals used for adaptive plan generation are listed in Table 1.

**Table 1:** Adaptive plan optimization goals. The GTV encompassed bilateral adrenal masses with 0 mm PTV. The conformality shell was a derived structure created as an automatic wall expansion around the GTV with a 0 cm inner margin and 2 cm outer margin and assisted with dose conformality between the bilateral targets. GTV, gross tumor volume

Organ	Goal	Acceptable variation	Priority
GTV	V2500 cGy ≥ 99%	≥95%	1
GTV	D0.03 cm^3^ < 105%	≤108%	1
Bowel	D0.03 cm^3^ ≤ 2,600 cGy	≤2,700 cGy	1
Spinal cord	D0.35 cm^3^ ≤ 2,200 cGy	-	1
Stomach	D0.03 cm^3^ ≤ 2,600 cGy	-	1
GTV	V2400 cGy ≥ 100%	≥97%	1
Conformality shell	D0.03 cm^3^ ≤ 2,500 cGy	-	1
Conformality shell	Dmean ≤ 1,700 cGy	≤1,800 cGy	1
Kidney_L	V1800 cGy ≤ 20 cm^3^	≤100 cm^3^	2
Kidney_R	V1800 cGy ≤ 20 cm^3^	≤100 cm^3^	2
Bowel	V2000 cGy ≤ 30 cm^3^	-	2
RenalHilum_L	V2300 cGy ≤ 5 cm^3^	≤15 cm^3^	2
RenalHilum_R	V2300 cGy ≤ 5 cm^3^	≤15 cm^3^	2
Kidneys	V1800 cGy ≤ 50 cm^3^	≤200 cm^3^	2
Spleen	Dmean ≤ 450 cGy	≤500 cm^3^	3
Pancreas	Dmean ≤ 900 cGy	≤1,000 cm^3^	3
Liver	V2100 cGy ≤ 400 cm^3^	≤700 cm^3^	3

The patient started adaptive RT sessions on day 33 and ended on day 12 with no significant side effects. The patient attended four RT sessions; however, RT was withheld at sessions 2 and 4 due to a significant reduction in tumor volume exceeding 10% as per the treatment protocol, resulting in a total delivery of 10 Gy.

Details of once-weekly adaptive sessions

Session 1

**Figure 2:**
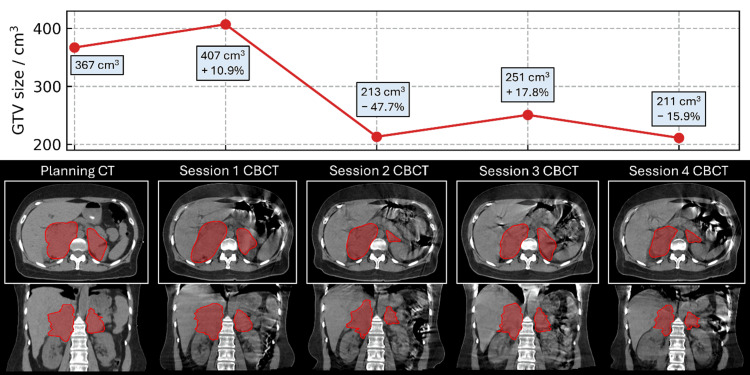
Progression of gross tumor volume (GTV) size throughout the course of treatment, where the percentage change for each timepoint is in reference to the previous session. The GTV contour is shown on the planning CT and the cone-beam CT (CBCT) for each of the four sessions. Treatments were delivered only for sessions 1 and 3.

Eight days passed between the CT simulation and the first fraction. The HyperSight cone-beam CT (CBCT) image quality was deemed sufficient for visualization and contouring despite some motion artifacts around the anterior abdomen and bowel gas (Figure 2). No motion management was used for imaging or during treatment delivery. The Ethos AI auto-contouring software generated right and left kidney, liver, stomach, and bowel contours on the CBCT. The AI-generated liver included the right adrenal mass, which was located adjacent to the liver and had a similar appearance on CT. The GTV and remaining organs (right and left renal hilum, pancreas, spinal cord, and spleen) were deformed from the planning CT to the CBCT, but the Ethos software cropped parts of the deformed GTV that overlapped with the erroneous AI liver contour, despite the GTV not being a derived structure. For future fractions, the liver was modified to exclude the GTV prior to generating the deformed GTV. The normal organ contours and the GTV were reviewed by a physician before optimizing the adapted plan. After selecting the adaptive treatment plan, another CBCT was acquired prior to treatment delivery to verify the patient's position and anatomy had not changed.

**Figure 3:**
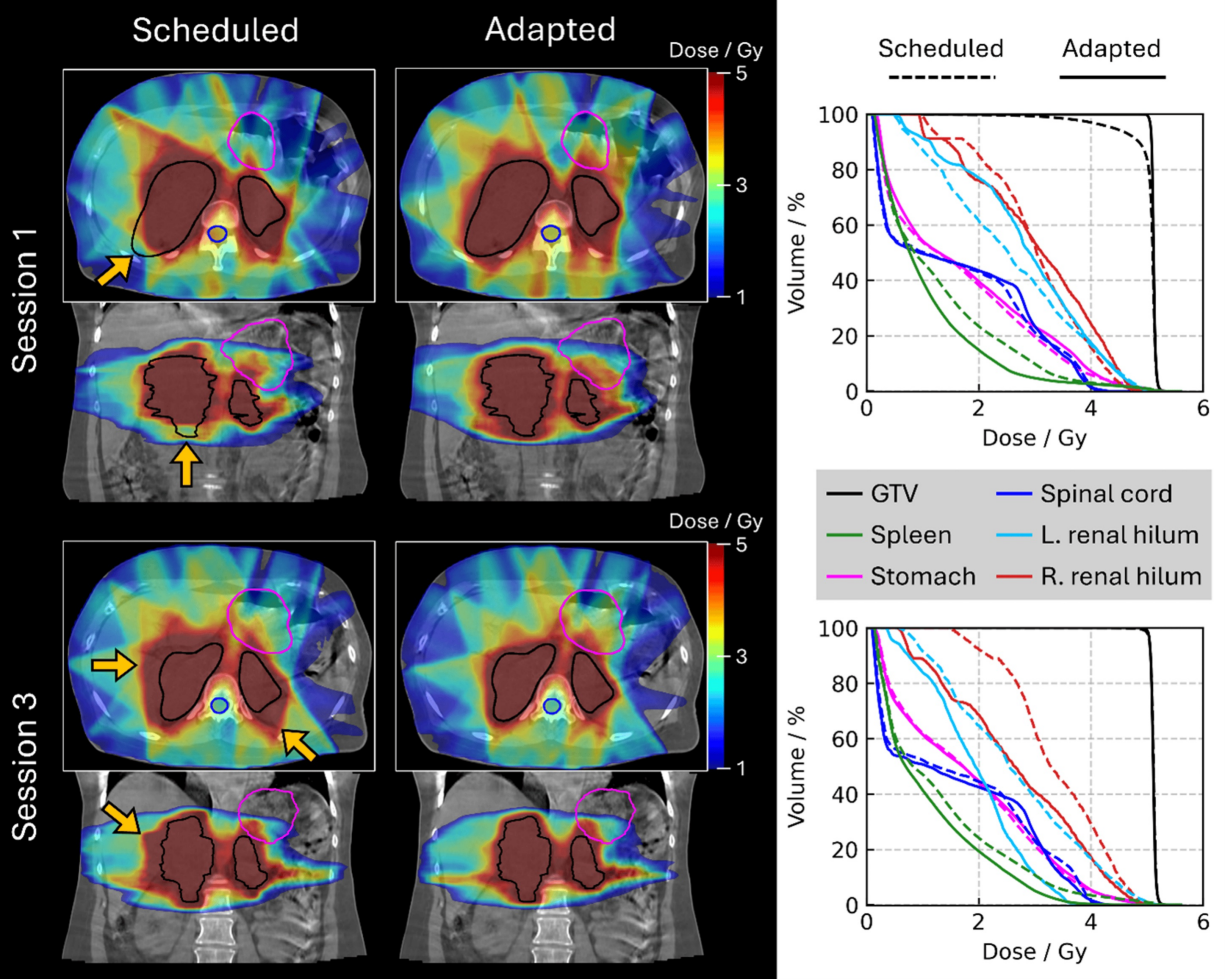
Comparison of the scheduled and adapted plans for the two sessions in which treatments were delivered. Arrows indicate where the GTV would have been underdosed when using the scheduled plan for session 1 and where the organs at risk (OARs) would have received excess doses when using the scheduled plan for session 3.

As per CBCT, the disease had increased in volume by 10.9% during this time (Figure 2). Adaptive radiotherapy resulted in better target coverage as compared to the scheduled plan, as shown in Figure 3, and the patient was treated with the adaptive plan (5 Gy).

Session 2

One week after session 2, the CBCT showed a dramatic decrease in disease volume of 47.7%. Treatment was held due to excellent response (0 Gy). The patient’s pain and fatigue were significantly improved and did not recur.

Session 3

Another week later, the CBCT showed a mild increase in disease volume relative to session 2, yet this remained significantly smaller than session 1. As such, adaptive radiotherapy was delivered (5 Gy), as shown in Figure 3.

Session 4

A week later (day 12), the patient’s CBCT showed a further decrease in disease volume. Treatment was held (0 Gy). This was the final session, as the patient’s CAR T-cells were ready.

An interim PET/CT scan was conducted on day 7, showing a reduced bulk of disease (Figure 1). The patient began lymphodepleting chemotherapy with fludarabine and cyclophosphamide. On day 0, lisocabtagene maraleucel (liso-cel) CAR T-cells were administered in an inpatient setting. Subsequently, the patient developed grade 2 cytokine release syndrome (CRS), which was managed with three doses of tocilizumab and one dose of siltuximab. She also experienced biphasic grade 2 immune effector cell-associated neurotoxicity syndrome (ICANS), with a recrudescence of symptoms upon tapering steroids, and was managed with dexamethasone 10 mg intravenously.

Adaptive treatment timing analysis

A timing analysis was performed for the four online adaptive sessions, and the results are shown in Table 2. Only the contouring time and total treatment time are relevant for the two sessions where treatment was held. For the two sessions where online adaptive treatment was delivered, both required approximately 30 minutes from the first CBCT acquisition until the end of treatment delivery.

**Table 2:** Timing analysis for the online adaptive treatments. For fractions 2 and 4, only the contouring time is shown as treatment was withheld for these sessions. Times are shown in minutes and seconds (mm:ss). The total treatment time also includes acquiring a verification cone-beam CT (CBCT) and evaluating the anatomy for changes on that CBCT prior to treatment. The beam-on time is estimated as the total monitor units (MU) divided by the maximum dose rate.

Fraction	Contouring	Optimization	Plan review and QA	Beam-on time	Total treatment time
1	15:26	1:12	3:00	06:05	30:36
2	20:39	-	-	-	22:13
3	13:47	1:16	2:51	07:04	30:01
4	17:45	-	-	-	19:19

## Discussion

This patient showcases the successful application of adaptive RT as bridging therapy prior to CAR T-cell therapy. The patient experienced rapid symptomatic relief, with no RT-related toxicities, while being treated at a lower total dose than she otherwise would have received with standard, non-adaptive RT. CBCT allowed decision-making based on response assessment of whether or not the patient should receive additional RT based on pre-specified criteria - the patient received RT at only half of the sessions due to excellent tumor shrinkage. Post-RT, pre-CAR T-cell PET/CT showed significant disease regression, compatible with partial response. The patient successfully underwent lymphodepleting chemotherapy and CAR T-cell therapy infusion after adaptive bridging RT.

The once-weekly RT sessions allowed this patient to undergo RT as bridging therapy, whereas otherwise, daily RT for two or more weeks would have been difficult for this patient due to the distance from the hospital. The alternative of systemic therapy bridging therapy likely would have had worse toxicity. Furthermore, the total treatment time per session, when being treated, was only half an hour, representing a modest increase over conventional radiation timeslots.

The adaptive plans were universally better than the scheduled plans. In high-grade B-cell lymphomas, which commonly have a fast-doubling time, we learned that the disease can have considerable growth between CT simulation and the first fraction over a period of eight days here. Nonetheless, adaptive RT allowed the necessary modification of the patient’s target volume to encompass the real-time extent of the disease while respecting plan optimization goals. At subsequent sessions, tumor response was easy to monitor on CBCT, and when additional adaptive RT was administered, sometimes certain organs at risk (OARs) were more with the adaptive plan, but the latter was still chosen due to better target coverage.

## Conclusions

The patient case presented demonstrates a potential new role for CT-based adaptive RT as bridging therapy for patients undergoing CAR T-cell therapy, showing the feasibility of real-time adaptive RT assisted by AI, lack of toxicities related to RT, and excellent disease response to relatively low doses of hypofractionated RT allowing the patient to safely get to CAR T infusion. As mentioned above, this patient was treated on a clinical trial, which is now closed to accrual. We expect the results of this adaptive RT trial to guide the future of adaptive RT in relapsed/refractory B-cell lymphomas.
